# Age of e-cigarette initiation in USA young adults: Findings from the Population Assessment of Tobacco and Health (PATH) study (2013–2017)

**DOI:** 10.1371/journal.pone.0261243

**Published:** 2021-12-13

**Authors:** Adriana Pérez, Meagan A. Bluestein, Arnold E. Kuk, Baojiang Chen

**Affiliations:** 1 Department of Biostatistics and Data Science, the University of Texas Health Science Center at Houston (UTHealth), School of Public Health, Austin, Texas, United States of America; 2 Michael & Susan Dell Center for Healthy Living, The University of Texas Health Science Center at Houston (UTHealth), School of Public Health, Austin, Texas, United States of America; Christiana Care/University of Delaware, UNITED STATES

## Abstract

**Introduction:**

There is a lack of research prospectively estimating the age of e-cigarette initiation in U.S. young adults.

**Methods:**

Secondary analysis of PATH young adults across 2013–2017 (waves 1–4) were conducted. We prospectively estimated age of initiation of: ever, past 30-day, and fairly regular e-cigarette use using weighted interval-censoring survival analyses. Interval-censoring Cox proportional hazard models adjusting for sex, race/ethnicity, and previous use of six other tobacco products (cigarettes, traditional cigars, filtered cigars, cigarillos, hookah, and smokeless tobacco) were fitted for each of the three e-cigarette initiation outcomes.

**Results:**

Among never e-cigarette users, by age 21, 16.8% reported ever use, 7.2% reported past 30-day use, and 2.3% reported fairly regular e-cigarette use. Males had increased risk of initiating ever, past 30-day, and fairly regular e-cigarette use at earlier ages compared to females. Hispanic young adults had increased risk of initiating ever and past 30-day e-cigarette use at earlier ages compared to Non-Hispanic White young adults. Previous use of other tobacco products before e-cigarette initiation increased the risk of an earlier age of e-cigarette initiation.

**Conclusion:**

Prevention and education campaigns should focus on young adults in order to alleviate the public health burden of initiating e-cigarette use at earlier ages.

## Introduction

E-cigarettes sprung into popularity in 2013–2014, and the National Adult Tobacco Survey estimated that among young adults aged 18–24, the prevalence of ever e-cigarette use was 35.8% and the prevalence of past 30-day e-cigarette use was 13.6% [1]. Additionally, our previous study of PATH young adults (18–24 year olds) who had never used e-cigarettes at wave 1 (2013–2014) found that after 1 year of follow-up (2014–2015), 14.6% of young adults had ever used e-cigarettes and 4.5% had used e-cigarettes in the past 30-days [2]. These incidences of e-cigarette initiation were higher than ever use (9.2%) and past 30-day use (2.5%) reported by youth [2]. Similarly, a different cross-sectional study of PATH reported e-cigarette use among youth (12–17 years old), young adults (18–24 years old) and adults (25+ years old) also found that across the first three waves of PATH (2013–2014, 2014–2015, and 2015–2016), young adults had the highest prevalence of ever e-cigarette use (51.9% at wave 3), past 12-month e-cigarette use (29.8% at wave 3), and past 30-day e-cigarette use (17.2% at wave 3) [3]. Reducing tobacco product use nationwide has been contingent on understanding the age of initiation in order to educate the public and implement interventions that can be tailored to people of particular ages before they first start trying tobacco products. Previously mentioned studies indicate that young adults have a higher prevalence of e-cigarette use and initiation compared to youth [1–3], but what is unknown is the age at which young adults are most vulnerable to initiate e-cigarettes among those who did not initiate e-cigarette use during youth.

Young adulthood represents a developmental period that is distinct from youth and older adulthood. The emerging adulthood hypothesis postulates that young adulthood is characterized by developmentally discrete features that define the transition into adulthood during the ages 18–25, which help to explain the higher rates of tobacco and substance use in this age group compared to other ages [4]. Many emerging adults are moving out of their family homes for the first time with novel freedoms and opportunities [4], and a desire to have a wide range of experiences before they settle into adult life, which can lead to experimentation with different substances [4], including e-cigarettes.

Recent studies have shown that e-cigarette use may lead to subsequent cigarette use [5–8], but research on the impact of cigarette and other tobacco product use on e-cigarette initiation is emerging [9–12]. Other studies have shown e-cigarettes are commonly used as part of dual or poly-tobacco product use patterns, with only 3.8% of tobacco users reporting that e-cigarettes were the single tobacco product they had used in the past 30-days among the PATH 2013–2014 adults (18+ years) [13]. A different nationally representative sample of adults 18 and older in 2014 found that 93% of past 30-day e-cigarette users had also used cigarettes in the past 30-days [10]. In addition, the 2017 National Health Interview Survey (NHIS) found that the most common dual tobacco use pattern reported by adults 18+ was cigarettes/e-cigarettes (30.1%) [14].

Furthermore, now that e-cigarettes are a dominant feature of the tobacco marketplace, it is important to understand outcomes of initiation among young adults because e-cigarettes may not have been available to them during youth. In addition, the efficacy of the Tobacco 21 Law that changed the minimum age of tobacco sale from 18 to 21 in December 2019 [15] can be evaluated with studies that use data collected before this law went into effect. Finally, the Master Settlement Agreement, which prohibited tobacco companies from marketing to those under the minimum age of tobacco sale, resulted in young adults becoming a critical target for tobacco industry marketing practices [16]. Given that young adults are the legal targets of the tobacco industry and that young adults have the highest prevalence of e-cigarette use [13], we sought to estimate the age of e-cigarette initiation among young adults, including ever, past 30-day, and fairly regular e-cigarette use overall, by, sex and by race/ethnicity. Furthermore, the effect of previous use of six other tobacco products was controlled for when estimating differences in the age of initiation by sex and by race/ethnicity for each e-cigarette outcome. By examining three e-cigarette use outcomes, we will better understand patterns of e-cigarette initiation among young adults.

## Methods

The PATH study used a four-stage stratified area probability sampling design to provide a nationally representative sample of U.S. youth and adults in 2013–2014 (wave 1) with measurements conducted annually with wave 2 in 2014–2015, wave 3 in 2015–2016, and wave 4 in 2016–2017. The full details about sampling methodology for the PATH study have been described previously with a summary provided here [17]. The civilian household population of individuals aged 12 and older in all 50 U.S. states was the target population, with 9,110 young adults (aged 18–24) completing wave 1 and a 74.0% response rate in the adult interview [17]. Written informed consent was obtained from all adult participants prior to participation [17]. In addition, when PATH wave 1 youth (12–17 years old) turn 18 in subsequent waves, they were invited to participate in the adult measurements. Youth who turn 18 in subsequent waves of PATH also had to be never users to e-cigarettes to be included in our analysis. This resulted in 1,061 participants entering the study at wave 2 and 1,102 participants entering the study at wave 3. This resulted in n = 7,360 (N = 25,454,121) young adults who were never e-cigarette users at their first wave of adult participation (waves 1–3; 2013–2016) were included in our analysis with e-cigarette outcomes followed-up longitudinally in waves 2–4 (2014–2017). This study is a secondary analysis of the PATH adult restricted-use datasets, which were accessed through the Inter-university Consortium for Political and Social Research (ICPSR) server where the data are located [18]. The original investigators of the PATH study obtained written informed consent for adult participants. The University of Texas Health Center at Houston granted institutional review board approval (HSC-SPH-17-0368).

### Measures

#### Outcomes

Three e-cigarette outcomes of interest are age of initiation of: (i) ever use, (ii) past 30-day use, and (iii) fairly regular use of e-cigarettes. In wave 1, PATH measured ever e-cigarette use with the question: “Have you ever used an e-cigarette, such as NJOY, Blu, or Smoking Everywhere, even one or two times?”. In waves 2–4, in the PATH adult measurements, this question was modified: “Have you ever used an electronic nicotine product, even one or two times? (Electronic nicotine products include e-cigarettes, e-cigars, e-pipes, e-hookahs, personal vaporizers, vape pens and hookah pens.)”. These questions were assumed to measure the same construct across waves. Response options at all waves included “yes”, “no”, and “don’t know”. Those who answered “don’t know” were excluded from the analysis. In waves 2–4, past 30-day e-cigarette use was measured with the question: “In the past 30 days, on how many days did you use an e-cigarette?”. Numeric response options included 0–30 days and participants were considered past 30-day users if they reported e-cigarette use on 1 or more days. The measure of fairly regular use was included as a subjective measure in order to capture participants who consider themselves “regular” users [19]. In wave 2, “fairly regular” use was measured with the question: “Have you ever used e-cigarettes fairly regularly?”. In waves 3 and 4, participants were instead asked: “Have you ever used electronic nicotine products fairly regularly?”. These questions were assumed to measure the same construct across waves. Response options for both questions included “yes”, “no”, and “don’t know”. Those who responded “don’t know” were excluded from the analysis.

#### Sex and race/ethnicity

PATH imputed sex, race and Hispanic ethnicity at wave 1 but not at waves 2 and 3 [17]. Answers to a question about participant sex classified participants as either males or females. Participant race was measured with the following categories: White race alone, Black race alone, Asian race alone, and other race (including multi-racial). Ethnicity categorized participants as either Hispanic or Non-Hispanic. Answers to race and ethnicity questions were combined to create race/ethnicity categories that are comparable to those in prior Surgeon General’s reports [1, 20], which include: Non-Hispanic White, Hispanic, Non-Hispanic Black, Non-Hispanic Other (Non-Hispanic Asian, multi-race, and other races).

#### Previous use of other tobacco products

PATH asked all participants about ever use (Have you ever used [tobacco product], even once or twice/one or two puffs?) of: cigarettes, large cigars, cigarillos, filtered cigars, hookah, and smokeless tobacco. We examined previous ever use of these six other tobacco products at the wave prior to initiation of ever, past 30-day, and fairly regular e-cigarette use to ensure that the other tobacco product use preceded e-cigarette initiation. Therefore, 3 variables were created for each of the six other tobacco products to represent use of that product prior to ever, past 30-day, and fairly regular e-cigarette use.

#### Age of initiation

PATH uses a derived variable for participant age at each wave in years because date of birth is not included in the restricted-use data [21]. PATH also uses a derived variable to represent the number of weeks between waves that young adults participate in [21]. Age of initiation of each e-cigarette use outcome was estimated by adding participants’ age at their first wave of adult PATH participation (waves 1–3) to the number of weeks between relevant subsequent waves (waves 2–4) when the outcome was first reported for those who became users or the last report of never use among those who did not report each outcome. Participant age was converted from years to weeks and was added to this second variable to give us a more precise estimate of participant age, rather than using age in years at the wave that the participant reported the outcome. A lower and an upper age bound for each outcome was calculated. For all participants, the lower age bound was the age at the last wave where they reported never use of each e-cigarette outcome. For participants who become users, the upper age bound reflects the age at the last wave that they reported non-use plus the number of weeks to the wave when they first report initiation. The upper age bound was censored for never users.

### Statistical analysis & data management

All statistical analyses incorporated the use of sampling weights and 100 balance repeated replicate weights to account for PATH’s complex survey design with Fay’s adjustment set to 0.3 to increase estimate stability [21]. Sampling weights were used according to each participants’ first wave of adult participation in PATH. Weighted frequencies and percentages are reported for categorical variables and weighted means and standard errors are reported for continuous variables. The distributions of the age of initiation with respect to the three e-cigarette use outcomes were estimated using weighted nonparametric interval-censored survival analysis [22–26] and the Turnbull non-parametric estimator for confidence intervals [27]. Hazard functions are reported as cumulative probability in percentages (i.e., cumulative incidence). Differences in age of initiation for each e-cigarette outcome by sex and race/ethnicity while controlling for previous use of six other tobacco products were estimated by fitting weighted interval-censored Cox proportional hazards regression models with a piecewise constant function as the baseline hazard function. Hazard ratios (HRs) and 95% confidence intervals (CIs) are reported. A type I error level of 0.05 was used to determine statistical significance for all two-sided statistical tests. Because there were differences in the age of initiation by sex and by race/ethnicity the hazard function was stratified by these variables. These hazard functions that show the full distribution of ages calculated within a week’s precision are displayed in figures. There was very little missingness in PATH, and the missing values are reported. SAS version 9.4-TSlevel1M6 was used to complete all statistical analyses [28].

## Results

Demographic characteristics for the PATH young adults who were never users of e-cigarettes at their first wave of adult participation (waves 1–3) are presented in Table 1. Among the 7,360 young adults, representing 25,454,121 young adults in the US who were never users of e-cigarettes, their average age was 20.44 (SE = 0.03) at their first wave of adult study participation, 53.6% were female, 51.7% were Non-Hispanic White, 21.0% were Hispanic, 15.6% were Non-Hispanic Black and 11.6% were Non-Hispanic Other. Before ever e-cigarette initiation, 32.3% of young adults reported previous cigarette use, and the proportions for the five other tobacco products are reported in Table 1. Previous use of the six other tobacco products prior to initiation of past 30-day and fairly regular e-cigarette use are included in S1 Table.

**Table 1:** Demographic characteristics of PATH^a^ USA young adult (aged 18–24) never e-cigarette users at their first wave of adult participation (2013–2016).

Variables		Never e-cigarette users at their first wave of adult participation	
		N^b^ = 7,360; N^b^ = 25,454,121	
		n (N)	weighted % (SE)
**Wave of entry into study**	Wave 1 young adult	5,197 (20,747,454)	81.5 (0.27)
	Wave 2 young adult	1,061 (2,350,329)	9.2 (0.18)
	Wave 3 young adult	1,102 (2,356,338)	9.3 (0.20)
**Age at entry into study**	weighted mean (SE)	20.44 (0.03)	
**Sex**	Male	3,363 (11,805,689)	46.4 (0.38)
	Female	3,994 (13,638,898)	53.6 (0.38)
	Missing	n = 3 (N = 9,534)	
**Race/Ethnicity**	Non-Hispanic White	3,457 (13,164,311)	51.7 (0.99)
	Hispanic	1,860 (5,342,006)	21.0 (0.54)
	Non-Hispanic Black	1,337 (3,966,756)	15.6 (0.75)
	Non-Hispanic Other	688 (2,940,758)	11.6 (0.68)
	Missing	n = 18 (N = 40,290)	
**Previous Use of Other Tobacco Products Before Ever E-cigarette Initiation**			
**Cigarettes**	Yes	2,739 (8,243,641)	32.3 (0.88)
	No	4,537 (17,018,190)	66.9 (0.87)
	Missing	n = 84 (N = 192,289)	
**Cigarillos**	Yes	1,969 (5,677,393)	22.3 (0.70)
	No	5,208 (19,272,590)	75.7 (0.74)
	Missing	n = 183 (N = 504,137)	
**Traditional Cigars**	Yes	993 (3,141,337)	12.3 (0.60)
	No	6,232 (21,942,098)	86.2 (0.61)
	Missing	n = 135 (N = 370,685)	
**Filtered Cigars**	Yes	664 (1,877,374)	7.4 (0.34)
	No	6,504 (23,058,139)	90.6 (0.40)
	Missing	n = 192 (N = 518,608)	
**Hookah**	Yes	2,609 (7,389,082)	29.0 (0.98)
	No	4,665 (17,866,562)	70.2 (0.97)
	Missing	n = 86 (N = 198,477)	
**Smokeless Tobacco**	Yes	543 (1,588,071)	6.2 (0.33)
	No	6,682 (23,548,524)	92.5 (0.35)
	Missing	n = 135 (N = 317,526)	

^a^: PATH restricted file received disclosure to publish on August 27, 2020 and April 13, 2021. United States Department of Health and Human Services. National Institutes of Health. National Institute on Drug Abuse, and United States Department of Health and Human Services. Food and Drug Administration. Center for Tobacco Products. Population Assessment of Tobacco and Health (PATH) Study [United States] Restricted-Use Files. ICPSR36231-v13. AnnArbor,MI: Inter-university Consortium for Political and Social Research [distributor], November 5, 2019. https://doi.org/10.3886/ICPSR36231.v23.

^b^: n = sample size; N = estimated population size.

**Fig 1:**
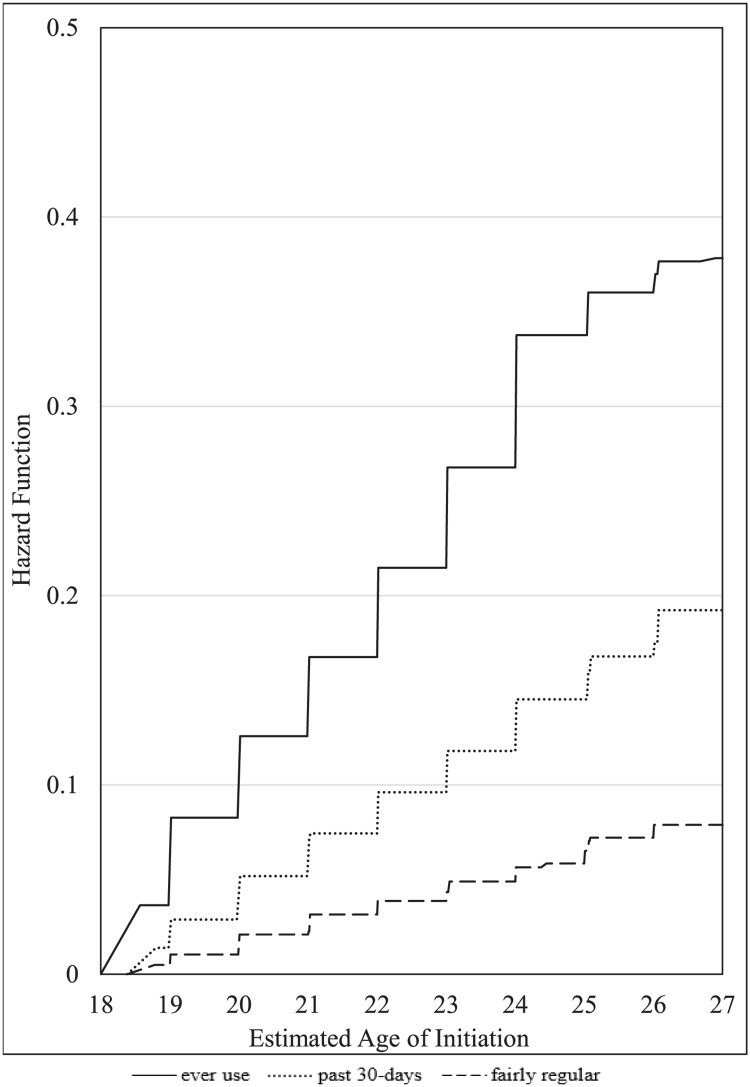
Estimated age of e-cigarette initiation in PATH young adults.

Table 2 shows the distribution of the estimated age of initiation for each of the three e-cigarette use outcomes overall, and the full distribution of ages within a week’s precision are displayed in Fig 1. We found that by age 21, 16.8%, 7.2%, and 2.3% were estimated to initiate ever use, past 30-day use, and fairly regular e-cigarette use, respectively. The highest increase in initiation of ever e-cigarette use occurs between 18 and 19 years old (8.3%), representing 2.2 million young adults.

**Table 2:** Estimated hazard function* of the age of initiation of e-cigarette outcomes and 95%CIs^a^ for the overall sample of PATH^¥^ USA young adults (aged 18–24).

Age	Ever e-cigarette use	Past 30-day e-cigarette use	Fairly regular e-cigarette use
**18**	0.0%	0.0%	0.0%
**19**	8.3% (7.5–9.1)	2.9% (2.0–3.8)	1.0% (0.0–2.1)
**20**	12.6% (11.6–13.6)	5.2% (3.9–6.5)	2.1% (1.4–2.8)
**21**	16.8% (15.5–18.0)	7.2% (4.3–10.2)	2.3% (0.7–3.9)
**22**	21.5% (19.9–23.0)	9.6% (7.2–12.1)	3.9% (3.2–4.6)
**23**	26.8% (25.1–28.5)	11.8% (9.5–14.1)	4.3% (2.9–5.8)
**24**	33.8% (31.0–36.6)	14.5% (11.2–17.8)	5.6% (4.5–6.8)
**25**	33.8% (29.6–38.0)	14.5% (11.5–17.6)	6.5% (4.7–8.3)
**26**	37.0% (33.7–40.3)	17.5% (15.1–19.8)	7.9% (6.3–9.5)
**27**	37.8% (35.1–40.6)	19.2% (17.3–21.1)	N/A

*Hazard functions are reported as cumulative percentages (i.e., cumulative incidence).

^a^: 95%CI: Turnbull 95% confidence interval.

N/A: not enough sample size to estimate initiation at that age.

^¥^ PATH restricted file received disclosure to publish: July 24, 2020. United States Department of Health and Human Services. National Institutes of Health. National Institute on Drug Abuse, and United States Department of Health and Human Services. Food and Drug Administration. Center for Tobacco Products. Population Assessment of Tobacco and Health (PATH) Study [United States] Restricted-Use Files. ICPSR36231-v13. AnnArbor,MI: Inter-university Consortium for Political and Social Research [distributor], November 5, 2019. https://doi.org/10.3886/ICPSR36231.v23.

Table 3 presents the results of the crude and adjusted Cox proportional hazard models. While adjusting for race/ethnicity and previous use of six other tobacco products before each e-cigarette initiation outcomes, males were at increased risk of initiating ever (AHR = 1.15; 95%C 1.02–1.28), past 30-day (AHR = 1.21; 95%CI = 1.02–1.45) and fairly regular e-cigarette use (AHR = 1.76; 95%CI = 1.30–2.39) at earlier ages compared to females.

**Table 3:** Crude and adjusted hazard ratios (95% confidence intervals) for the age of e-cigarette initiation outcomes among PATH^a^ young adults (2013–2017).

Variables		Ever	Past 30-day	Fairly regular
		Crude Hazard Ratios		
**Sex** ^b^	Male	**1.19 (1.07–1.34)**	**1.29 (1.09–1.52)**	**1.89 (1.44–2.48)**
**Race/ethnicity** ^c^	Hispanic	**1.38 (1.21–1.57)**	**1.29 (1.07–1.55)**	0.87 (0.65–1.16)
	Non-Hispanic Black	1.11 (0.92–1.32)	0.93 (0.73–1.19)	**0.57 (0.37–0.86)**
	Non-Hispanic Other^d^	0.84 (0.64–1.09)	0.88 (0.60–1.28)	**0.66 (0.45–0.95)**
**Previous use of other tobacco products before e-cigarette initiation use outcomes**^e^ **(waves 1–3)**	Cigarettes	**2.00 (1.78–2.26)**	**2.01 (1.70–2.36)**	**2.61 (2.04–3.33)**
	Cigarillos	**2.22 (1.96–2.52)**	**2.10 (1.77–2.48)**	**2.07 (1.64–2.61)**
	Traditional Cigars	**1.30 (1.12–1.51)**	**1.56 (1.31–1.85)**	**2.05 (1.60–2.63)**
	Filtered Cigars	**2.00 (1.73–2.30)**	**2.17 (1.79–2.63)**	**2.62 (2.01–3.40)**
	Hookah	**2.22 (1.95–2.53)**	**1.96 (1.70–2.26)**	**1.99 (1.54–2.57)**
	Smokeless Tobacco	**1.71 (1.45–2.02)**	**1.60 (1.26–2.02)**	**2.18 (1.54–3.08)**
**Variable**	**Adjusted Hazard Ratios** ^f^			
**Sex** ^b^	Male	**1.15 (1.02–1.28)**	**1.21 (1.02–1.45)**	**1.76 (1.30–2.39)**
**Race/ethnicity** ^c^	Hispanic	**1.31 (1.15–1.50)**	**1.24 (1.03–1.49)**	0.83 (0.59–1.18)
	Non-Hispanic Black	1.02 (0.86–1.21)	0.88 (0.69–1.12)	**0.66 (0.45–0.96)**
	Non Hispanic Other^d^	0.95 (0.74–1.21)	1.01 (0.70–1.48)	**0.60 (0.38–0.95)**
**Previous use of other tobacco products before e-cigarette initiation use outcomes**^e^ **(waves 1–3)**	Cigarettes	**1.34 (1.18–1.53)**	**1.40 (1.14–1.72)**	**1.98 (1.45–2.71)**
	Cigarillos	**1.60 (1.41–1.82)**	**1.47 (1.16–1.86)**	1.08 (0.81–1.45)
	Traditional Cigars	**0.68 (0.59–0.79)**	0.86 (0.70–1.05)	0.98 (0.71–1.34)
	Filtered Cigars	**1.26 (1.07–1.49)**	**1.42 (1.11–1.82)**	**1.55 (1.15–2.08)**
	Hookah	**1.74 (1.52–2.00)**	**1.48 (1.24–1.77)**	1.39 (1.00–1.92)
	Smokeless Tobacco	1.14 (0.93–1.40)	0.95 (0.73–1.24)	0.94 (0.62–1.43)

^a^: PATH restricted file received disclosure to publish: April 13, 2021. United States Department of Health and Human Services. National Institutes of Health. National Institute on Drug Abuse, and United States Department of Health and Human Services. Food and Drug Administration. Center for Tobacco Products. Population Assessment of Tobacco and Health (PATH) Study [United States] Restricted-Use Files. ICPSR36231-v13. AnnArbor,MI: Inter-university Consortium for Political and Social Research [distributor], November 5, 2019. https://doi.org/10.3886/ICPSR36231.v23.

^b^: The reference category for sex is females.

^c^: The reference category for race/ethnicity is Non-Hispanic White.

^d^:Non-Hispanic other includes Asian, multi-race, etc.

^e^: The reference category for each of the tobacco products previously used before e-cigarette initiation is “no”.

^f^: Each model is adjusted for sex, race/ethnicity, and previous use of other tobacco products before each e-cigarette initiation outcome (i.e., cigarettes, cigarillos, traditional cigars, filtered cigars, hookah, and smokeless tobacco).

While adjusting for sex and previous use of six other tobacco products before each e-cigarette initiation outcome, Hispanic young adults were at increased risk of initiating ever (AHR = 1.31; 95%CI = 1.15–1.50) and past 30-day e-cigarette use (AHR = 1.24; 95%CI = 1.03–1.49) at earlier ages compared to Non-Hispanic White young adults. Adjusted models revealed that Non-Hispanic Black young adults (AHR = 0.66; 95%CI = 0.45–0.96) and Non-Hispanic other young adults (AHR = 0.54; 95%CI = 0.34–0.85) were less likely to initiate fairly regular e-cigarette use at earlier ages compared to Non-Hispanic White young adults.

**Fig 2:**
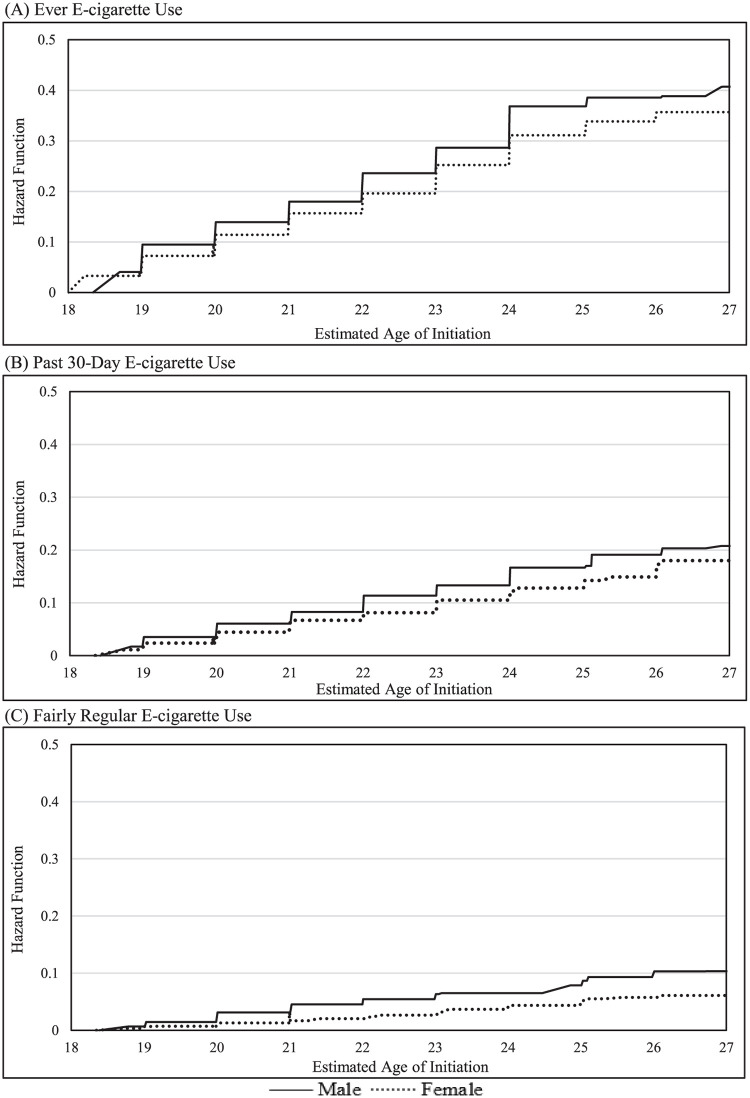
Estimated age of e-cigarette initiation in PATH young adults stratified by sex.

**Fig 3:**
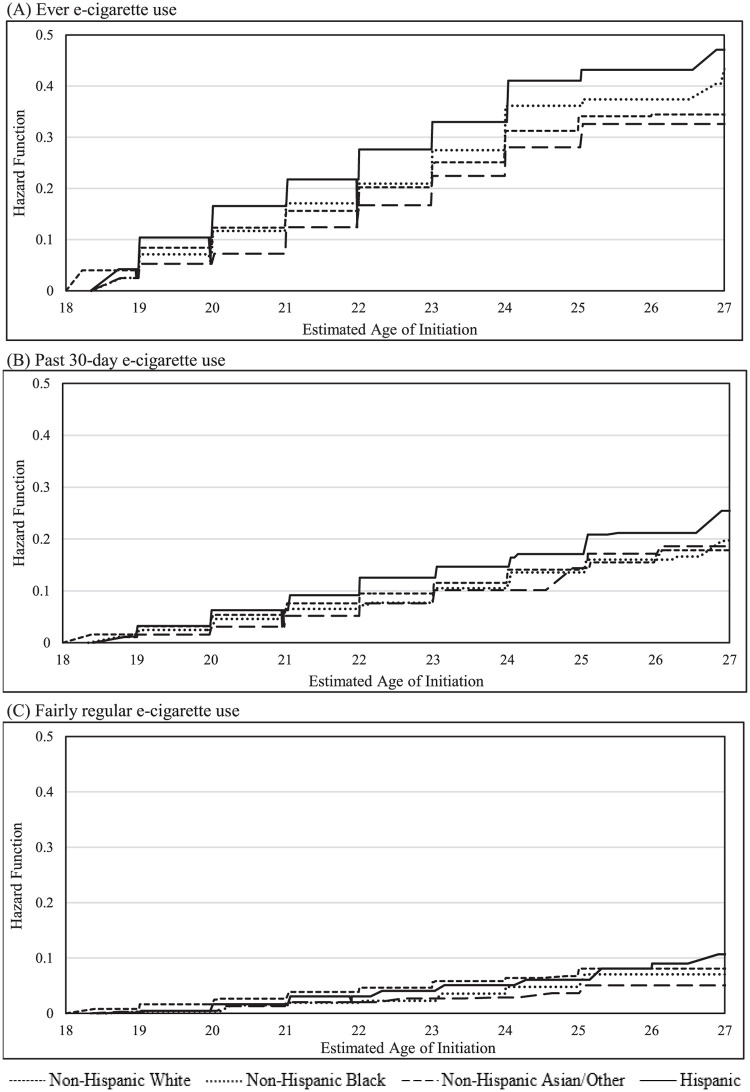
Estimated age of e-cigarette initiation in PATH young adults stratified by race/ethnicity.

When adjusting for sex and race/ethnicity, previous ever use of cigarettes, cigarillos, filtered cigars, and hookah were associated with an increased risk of an earlier age of ever e-cigarette initiation. When adjusting for sex and race/ethnicity, previous ever use of cigarettes, cigarillos, filtered cigars, and hookah were associated with an increased risk for an earlier age of past 30-day e-cigarette initiation. Finally, when adjusting for sex and race/ethnicity, previous ever use of cigarettes and filtered cigars were associated with an increased risk for an earlier age of fairly regular e-cigarette initiation. The distributions of the age of initiation of e-cigarette outcomes stratified by sex and by race/ethnicity are presented in Table 4, and the full distribution of ages within a week’s precision are displayed in Figs 2 and 3. We found that by age 25, the upper limit of young adulthood, 36.8% of males and 31.1% of females were estimated to initiate ever e-cigarette use. The largest increases in initiation of ever e-cigarette use occurred between ages 18 and 19 for males and females (9.5% and 7.2%, respectively). By age 25, 16.7% of males and 12.8% of females were estimated to initiate past 30-day e-cigarette use. The largest increase in initiation of past 30-day e-cigarette use occurred between ages 21 and 22 (5.3%) for males and between ages 25 and 26 for females (4.5%). By age 25, 7.9% of males and 5.2% of females were estimated to initiate fairly regular e-cigarette use.

**Table 4:** Estimated hazard functions^a^ and 95%CIs^a^ of the age of e-cigarette initiation outcomes for PATH^b^ USA young adults by sex and by race/ethnicity.

Age	Sex		Race/Ethnicity			
	Male	Female	Non-Hispanic White	Hispanic	Non-Hispanic Black	Non-Hispanic Other^c^
**Initiation of ever e-cigarette use**						
**18**	0.0%	0.0%	0.0%	0.0%	0.0%	0.0%
**19**	9.5% (7.7–11.3)	7.2% (6.3–8.2)	8.4% (7.1–9.8)	10.4% (8.7–12.1)	7.1% (5.4–8.8)	5.3% (3.1–7.5)
**20**	13.9% (12.6–15.3)	11.4% (10.2–12.8)	12.4% (10.8–13.9)	16.6% (14.6–18.6)	11.7% (9.6–13.9)	7.2% (4.7–9.8)
**21**	18.0% (14.3–21.7)	15.7% (9.7–21.7)	15.6% (10.4–20.8)	16.7% (4.8–28.4)	17.1% (11.1–23.0)	12.4% (9.2–15.7)
**22**	23.6% (21.3–25.9)	19.6% (17.9–21.4)	20.2% (17.7–22.7)	27.6% (24.4–30.9)	21.0% (17.4–24.5)	16.7% (9.3–24.1)
**23**	28.6% (26.0–31.2)	25.2% (22.9–27.5)	25.0% (19.8–30.2)	33.0% (23.3–42.7)	27.5% (23.1–31.8)	22.5% (17.4–27.6)
**24**	36.8% (31.3–42.4)	31.1% (24.8–37.4)	31.3% (26.9–35.6)	33.0% (28.8–37.2)	36.1% (26.6–45.6)	28.1% (17.8–38.3)
**25**	36.8% (33.0–40.7)	31.1% (28.4–33.8)	33.8% (28.0–39.6)	41.1% (37.3–44.9)	36.2% (30.9–41.4)	28.1% (20.7–35.4)
**26**	3u.6% (35.3–41.9)	35.7% (31.5–39.9)	34.5% (30.9–38.0)	43.2% (39.0–47.4)	37.4% (32.4–42.4)	32.6% (23.9–41.3)
**27**	40.7% (36.9–44.5)^t^	35.7% (32.2–39.2)	35.9% (30.9–40.9)^i^	N/A	44.0% (35.8–52.2)	N/A
**Initiation of past 30-day e-cigarette use**						
**18**	0.0%	0.0%	0.0%	0.0%	0.0%	0.0%
**19**	3.5% (1.8–5.2)	2.4% (1.9–2.8)	3.2% (2.2–4.1)	3.3% (2.0–4.5)	2.4% (0.0–5.0)	1.6% (0.5–2.7)
**20**	6.1% (3.6–8.5)	4.4% (3.6–5.3)	4.9% (1.2–8.6)	6.3% (4.2–8.3)	4.6% (1.8–7.4)	3.1% (1.0–5.2)
**21**	6.1% (5.0–7.1)	6.7% (3.5–9.9)	7.6% (6.3–8.9)	9.2% (6.4–11.9)	6.5% (3.6–9.5)	5.2% (1.0–9.4)
**22**	11.4% (10.0–12.8)	8.1% (5.9–10.4)	9.5% (6.9–12.1)	12.6% (7.5–17.6)	7.2% (5.0–9.5)	7.6% (4.7–10.5)
**23**	13.3% (11.7–14.9)	10.5% (8.7–12.3)	11.5% (9.2–13.9)	14.7% (9.9–19.4)	10.5% (8.2–12.8)	10.1% (6.4–13.8)
**24**	16.7% (13.6–19.8)	12.3% (8.8–15.7)	14.1% (10.7–17.5)	16.4% (10.4–22.4)	11.6% (7.8–15.4)	10.1% (6.0–14.3)
**25**	16.7% (14.7–18.6)	12.8% (11.1–14.5)	14.1% (10.8–17.3)	20.8% (12.7–29.0)	13.6% (8.9–18.2)	14.4% (8.1–20.6)
**26**	19.1% (16.8–21.5)	17.3% (13.4–21.2)	17.0% (14.0–19.9)	21.2% (15.6–26.8)	16.0% (12.3–19.8)	18.6% (10.2–27.0)
**27**	20.8% (17.5–24.1)	18.0% (15.2–20.7)	17.8% (15.4–20.2)	N/A	19.8% (14.4–25.1)	18.6% (10.7–26.5)
**Initiation of fairly regular e-cigarette use**						
**18**	0.0%	0.0%	0.0%	0.0%	0.0%	0.0%
**19**	0.7% (0.3–1.0)	0.7% (0.0–1.3)	1.6% (0.1–3.2)	0.5% (0.0–0.9)	0.4% (0.0–0.8)	0.3% (0.0–1.3)
**20**	3.1% (1.8–4.5)	1.3% (0.1–2.4)	2.5% (0.7–4.3)	1.6% (0.8–2.5)	0.4% (0.0–0.8)	1.3% (0.3–2.3)
**21**	3.1% (2.4–3.9)	1.7% (0.7–2.7)	3.9% (3.1–4.6)	1.6% (0.8–2.5)	1.9% (0.9–3.0)	2.0% (0.8–3.3)
**22**	5.4% (4.4–6.5)	2.0% (1.2–2.9)	4.6% (3.7–5.6)	3.1% (1.8–4.3)	2.3% (1.2–3.3)	2.0% (0.8–3.2)^f^
**23**	6.3% (4.1–8.6)	2.6% (1.8–3.5)	5.7% (3.4–7.9)	5.1% (3.4–6.8)	3.6% (2.1–5.1)	2.7% (1.1–4.2)^h^
**24**	6.5% (5.4–7.5)	4.4% (3.3–5.4)	6.4% (4.7–8.1)	6.1% (3.7–8.4)^d^	4.8% (2.5–7.1)	2.9% (1.3–4.4)
**25**	7.9% (3.7–9.0)	5.2% (3.0–7.4)	8.1% (5.5–10.7)	6.1% (4.3–7.8)	7.1% (3.1–11.0)	5.1% (2.8–7.3)
**26**	10.3% (7.8–12.8)	5.7% (4.0–7.5)	8.1% (6.4–9.8)	9.0% (5.6–12.5)	7.1% (4.0–10.1)	5.1% (2.7–7.4)
**27**	N/A	N/A	N/A	N/A	N/A	N/A

^a^: Hazard functions are reported as cumulative percentages (i.e., cumulative incidence); 95%CIs: Turnbull 95% confidence intervals.

^b^: PATH restricted file received disclosure to publish: July 13, 2020. United States Department of Health and Human Services. National Institutes of Health. National Institute on Drug Abuse, and United States Department of Health and Human Services. Food and Drug Administration. Center for Tobacco Products. Population Assessment of Tobacco and Health (PATH) Study [United States] Restricted-Use Files. ICPSR36231-v13. AnnArbor, MI: Inter-university Consortium for Political and Social Research [distributor], November 5, 2019. https://doi.org/10.3886/ICPSR36231.v23.

^c^: Non-Hispanic Other includes Asian, multi-race, etc.

We estimated that by age 25, 33.8%, 41.1%, 36.2%, and 28.1% of Non-Hispanic White, Hispanic, Non-Hispanic Black, and Non-Hispanic other young adults initiated ever e-cigarette use. The highest increase in ever e-cigarette initiation occurred between ages 18 and 19 for Non-Hispanic White (8.4%), ages 21 and 22 for Hispanic (10.9%), ages 23 and 24 for Non-Hispanic Black (8.6%), and ages 22 and 23 (5.8%) for Non-Hispanic other young adults. By age 25, 14.1%, 20.8%, 13.6%, and 14.4% of Non-Hispanic White, Hispanic, Non-Hispanic Black, and Non-Hispanic other young adults initiated past 30-day e-cigarette use. The largest increase in past 30-day e-cigarette initiation occurred between ages 20 and 21 for Non-Hispanic White (2.7%), ages 24 and 25 for Hispanic (4.4%), ages 26 and 27 for Non-Hispanic Black (3.8%), and ages 25 and 26 for Non-Hispanic Other young adults (4.3%). By age 25, 8.1%, 6.1%, 7.1% and 5.1% of Non-Hispanic White, Hispanic, Non-Hispanic Black, and Non-Hispanic other young adults initiated fairly regular e-cigarette use.

## Discussion

This is the first study to prospectively estimate the age of e-cigarette initiation among young adults who were never users of e-cigarettes using survival analyses in a nationally representative sample. We found that there was a substantial increase in initiation throughout young adulthood. E-cigarettes did not enter the U.S. market until 2007 and did not become the most widely used tobacco product until 2013 [1], so the PATH sample of young adults is uniquely situated to explore e-cigarette initiation in young adulthood since these products were not widely available to them during adolescence.

Several previous PATH studies have reported the prevalence of e-cigarette initiation in young adults. For example, a 2013–2015 PATH study of young adult (18–24 years old at wave 1) never e-cigarette users found after 1 year of follow-up that 14.6% had initiated ever e-cigarette use and 4.5% had initiated past 30-day e-cigarette use [2]. In addition, a different PATH study of young adult never users of any tobacco product at 2013–2014 or 2014–2015 reported 28.4% and 7.4% initiated past 12-month and past 30-day e-cigarette initiation [29]. Another study of PATH young adults (ages 18–24 at wave 1) found that ever e-cigarette use increased from 32.1% in 2013–2014 to 51.9% in 2015–2016 and that past 30-day e-cigarette use increased from 12.5% in 2013–2014 to 17.2% to 2015–2016 [3]. They also found that both types of e-cigarette use were highest among young adults compared to youth and older adults (25+) [3]. The difference between the current study and these other studies of PATH is that the first and second only includes 1 year of follow-up [2], the third includes ever and past 30-day e-cigarette users at each wave cross-sectionally, while we examine these outcomes prospectively [3], and our study focuses on the age of initiation instead of just the e-cigarette use outcomes.

While many studies have found that the prevalence of e-cigarette use is higher in young adults (18–24 years old) compared to youth (12–17 years old) [1–3], what was previously unreported was the age of initiation of e-cigarette use in a prospective longitudinal study among youth and young adult never e-cigarette users. We found in a previous study of PATH youth never e-cigarette users (12–17 years old at waves 1, 2, or 3 and followed-up through waves 2–4: 2014–2017), that by age 18, 41.7% reported initiation of ever e-cigarette use, 23.5% reported initiation of past 30-day e-cigarette use, and 10.3% reported initiation of fairly regular e-cigarette use [30]. These incidence rates of initiation are actually higher than our current findings among PATH young adults that followed-up participants across the same period of time. This is in contrast to the previously mentioned studies of PATH that found that e-cigarette initiation was higher among young adults compared to youth either cross-sectionally or after 1 year of follow-up [3]. While e-cigarette initiation seems to be higher in youth, there is still substantial initiation occurring in young adulthood that needs to be prevented.

We found consistently across 3 outcomes, that the age of e-cigarette initiation was younger in males than females. It is a well-established finding that young adult males have increased odds and higher prevalence of e-cigarette use [12, 31, 32], but our study takes this a step further by finding that young adult males initiate ever, past 30-day, and fairly regular e-cigarette use at earlier ages compared to females.

Previous research has found mixed evidence for differences in e-cigarette use by race/ethnicity. A study of college students in 2013 found that Non-Hispanic other college students had decreased odds of ever e-cigarette use (AOR = 0.46; 95%CI = 0.30–0.70), while there was no difference between Hispanic and Non-Hispanic White college students [12]. In addition, a previous 2013 study using a convenience sample of young adults found that there was no difference in past 30-day e-cigarette use between White and non-White young adults (AOR = 0.62; 95%CI = 0.20–1.86) [32]. These findings are in contrast to our study, which found that Hispanic young adults had increased risk of an earlier age of e-cigarette initiation compared to Non-Hispanic White young adults.

In our previous study of PATH youth (12–17 years) estimating the age of e-cigarette initiation prospectively, we found that Non-Hispanic Black and Non-Hispanic Other youth had decreased risk of initiating ever, past 30-day, and fairly regular e-cigarette use at earlier ages, while there was no difference between Hispanic and Non-Hispanic White youth in the age of ever e-cigarette initiation [30]. In contrast, this work found that Hispanic young adults have increased risk of initiating e-cigarettes at earlier ages compared to Non-Hispanic White young adults, while there was no difference for Non-Hispanic Black and Non-Hispanic Other. The findings in this study and our previous study provide indirect evidence for a difference in how race/ethnicity impacts the risk for initiating e-cigarettes at earlier ages between youth and young adults. Other studies have indicated that race/ethnicity differences in tobacco use during adolescence, in which Non-Hispanic Whites have the highest prevalence compared to other racial/ethnic groups, disappear during young adulthood (i.e., the age crossover hypothesis) [33, 34] and that could explain these differences in findings. More research is needed across national study samples to determine the risk factors that contribute to e-cigarette initiation in young adulthood.

Some important findings from this paper are that previous use of other tobacco products before e-cigarette initiation was associated with age of initiation of e-cigarette outcomes when adjusted for sex and race/ethnicity. Tobacco product use is a modifiable behavior, and our findings suggest that comprehensive prevention and cessation programs that address the use of all tobacco products are needed in young adults in order to reduce e-cigarette initiation Multiple tobacco product use is particularly common among e-cigarette users, with 55.9% of past 30-day e-cigarette users in a nationally representative sample of 13–25 year olds reporting that they also use other tobacco products [35]. A different study found that multiple tobacco product use is less common when the age of first tobacco product use is delayed (AOR = 0.89; 95%CI = 0.80–0.98) [36]. This study goes beyond previous evidence by finding previous use of other tobacco products, cigarettes and filtered cigars in particular, predisposed young adults toward younger ages of e-cigarette initiation among young adults who had previously never used e-cigarettes. These findings are concerning given that current use of multiple tobacco products can increase nicotine exposure and nicotine dependence [37, 38].

Taken together, the current study shows that all young adults could benefit from not only cessation programs, but prevention and education programs since initiation of e-cigarette use for the first time still occurs during young adulthood among those who had not initiated e-cigarette use during adolescence.

### Strengths and limitations

The primary strengths of our study are the use of PATH, a nationally representative dataset with national implications, the ability to prospectively estimate the age of e-cigarette initiation between 2013–2017 and estimating the age of initiation of e-cigarette initiation outcomes, which is more efficient than just reporting the prevalence of e-cigarette use. One limitation of the current study is that our oldest participants (24 years old at wave 1) would not have been exposed to e-cigarettes during youth, which would prohibit their ability to initiate e-cigarette use during youth. However, we wanted to examine e-cigarette initiation in young adults specifically. Another limitation is that it is not feasible to ask participants the exact date that they initiated e-cigarette use and the recall age of initiation suffers from recall bias [39, 40], so we estimated the age of initiation with interval-censoring survival methods. Identifying other risk and protective factors (e.g., education and employment status, peer pressure, work exposure or neighborhood exposure) answers a different research question, which is beyond the scope of this manuscript and requires additional considerations. The questions for peer influences in young adults were not asked in PATH until waves 3 and 4 and the PATH study does not allow for estimation by state or neighborhood areas, so we could not control for these variables in our analyses.

## Conclusion

This paper provides strong evidence for the age of e-cigarette initiation in young adulthood. In particular we found that males, Hispanic young adults, ever cigarette users, and those who have used other tobacco products before e-cigarette initiation are at increased risk of initiating e-cigarette use at earlier ages. Given that our research was conducted across years before the Tobacco 21 law was implemented, our findings show that a substantial amount of e-cigarette use could be prevented by changing the minimum age of tobacco sales. Now that the minimum age of tobacco sale has been increased to 21, future studies should use the current study as a comparison for measuring the efficacy of this law.

## Supporting information

S1 Table Previous use of other tobacco products before past 30-day and fairly regular e-cigarette initiation. (PDF)
