# Supplementary material for: Age of e-cigarette initiation in USA young adults: Findings from the Population Assessment of Tobacco and Health (PATH) study (2013–2017)
Source: PLoS One. 2021 Dec 13;16(12):e0261243. doi: 10.1371/journal.pone.0261243 (PMC8668126; doi:10.1371/journal.pone.0261243)
Supplement: S1 Table — (PDF) [file pone.0261243.s001.pdf]

Supplementary Table 1. Previous use of other tobacco products before past 30-day and fairly regular e-cigarette initiation.

Variables		Never e-cigarette users at first wave of adult participation	
		n= 7,360; N= 25,454,121	
		**n (N)**	**weighted % (SE)**
**Previous Tobacco Use Before Past 30-day E-cigarette Initiation**			
**Cigarettes**	Yes	2,837 (8,589,304)	33.7 (0.89)
	No	4,439 (16,672,527)	65.5 (0.91)
	Missing	84 (192,289)	
**Cigarillos**	Yes	2,067 (5,989,655)	23.5 (0.73)
	No	5,118 (18,979,255)	74.6 (0.77)
	Missing	175 (485,211)	
**Traditional Cigars**	Yes	1,144 (3,581,405)	14.1 (0.62)
	No	6,082 (21,503,780)	84.5 (0.64)
	Missing	134 (368,936)	
**Filtered Cigars**	Yes	750 (2,101,095)	8.3 (0.37)
	No	6,421 (22,840,228)	89.7 (0.43)
	Missing	189 (512,797)	
**Hookah**	Yes	2,742 (7,940,771)	31.2 (1.0)
	No	4,532 (17,314,873)	68.0 (1.0)
	Missing	86 (198,477)	
**Smokeless Tobacco**	Yes	566 (1,660,577)	6.5 (0.33)
	No	6,659 (23,476,018)	92.2 (0.35)
	Missing	135 (317,526)	
**Previous Tobacco Use Before Fairly Regular E-cigarette Initiation**			
**Cigarettes**	Yes	2,866 (8,719,808)	34.3 (0.92)
	No	4,410 (16,542,024)	65.0 (0.93)
	Missing	84 (192,289)	
**Cigarillos**	Yes	2,095 (6,073,673)	23.9 (0.74)
	No	5,090 (18,893,945)	74.2 (0.78)
	Missing	175 (486,503)	
**Traditional Cigars**	Yes	1,182 (3,697,648)	14.5 (0.64)
	No	6,044 (21,387,537)	84.0 (0.66)
	Missing	134 (368,936)	
**Filtered Cigars**	Yes	783 (2,188,986)	8.6 (0.40)
	No	6,389 (22,755,074)	89.4 (0.45)
	Missing	188 (510,061)	
**Hookah**	Yes	2,772 (8,086,257)	31.8 (1.1)
	No	4,502 (17,169,387)	67.5 (1.0)
	Missing	86 (198,477)	
**Smokeless Tobacco**	Yes	570 (1,669,892)	6.6 (0.34)
	No	6,655 (23,466,703)	92.2 (0.35)
	Missing	135 (317,526)	
